# The Chromatin Remodeler Brg1 Integrates ROS Production and Endothelial-Mesenchymal Transition to Promote Liver Fibrosis in Mice

**DOI:** 10.3389/fcell.2019.00245

**Published:** 2019-10-23

**Authors:** Zilong Li, Baoyu Chen, Wenhui Dong, Ming Kong, Yang Shao, Zhiwen Fan, Liming Yu, Dongmei Wu, Jun Lu, Junli Guo, Yong Xu

**Affiliations:** ^1^Key Laboratory of Biotechnology on Medical Plants of Jiangsu Province and School of Life Sciences, Jiangsu Normal University, Xuzhou, China; ^2^College of Health Sciences, Jiangsu Normal University, Xuzhou, China; ^3^Key Laboratory of Targeted Intervention of Cardiovascular Disease and Collaborative Innovation Center for Cardiovascular Translational Medicine, Department of Pathophysiology, Nanjing Medical University, Nanjing, China; ^4^Institute of Biomedical Research, Liaocheng University, Liaocheng, China; ^5^Cardiovascular Disease and Research Institute, Affiliated Hospital of Hainan Medical University, Haikou, China

**Keywords:** EndMT, ROS, liver fibrosis, transcriptional regulation, epigenetics

## Abstract

Trans-differentiation of endothelial cells to myofibroblast contributes to liver fibrosis. Reactive oxygen species (ROS) plays a key role in endothelial-mesenchymal transition (EndMT) although the underlying epigenetic mechanism is unclear. Here we report that endothelial conditional knockout of Brg1, a chromatin remodeling protein, attenuated liver fibrosis in mice. Brg1 deficiency in endothelial cells was paralleled by a decrease in ROS production and blockade of EndMT both *in vivo* and *in vitro*. The ability of BRG1 to regulate ROS production and EndMT was abolished by NOX4 depletion or inhibition. Further analysis revealed that BRG1 interacted with SMAD3 and AP-1 to mediate TGF-β induced NOX4 transcription in endothelial cells. Mechanistically, BRG1 recruited various histone modifying enzymes to alter the chromatin structure surrounding the NOX4 locus thereby activating its transcription. In conclusion, our data uncover a novel epigenetic mechanism that links NOX4-dependent ROS production to EndMT and liver fibrosis. Targeting the BRG1-NOX4 axis may yield novel therapeutics against liver fibrosis.

## Introduction

Liver fibrosis is considered a host defense mechanism in response to various injurious stimuli including trauma, ischemia, toxins, excessive nutrients, cholestasis, and pathogens (Hernandez-Gea and Friedman, 2011). Normally, resolution of fibrogenic response is accompanied by restoration of hepatic structure and function. Dysregulated liver fibrosis, however, is associated with disruption of liver anatomy and consequently loss of key liver functions typically seen in patients with end-stage liver diseases (Seki and Schwabe, 2015). Regardless of the specific etiologies, architectural and functional alterations observed in liver fibrosis are invariably synonymous with activation of myofibroblasts, the major source of extracellular matrix protein production and deposition (Duffield et al., 2013). The origins of myofibroblasts in fibrotic livers remain a hotly debated subject matter despite extensive research efforts invested with a body of literature. It has been proposed that various cell types, including epithelial cells, hepatic stellate cells (HSC), macrophages, and cholangiocyte may trans-differentiate into myofbriblast and thus contribute to liver fibrosis although the latest fate-mapping study seems to argue that HSCs are the predominant source of hepatic myofibroblast (Mederacke et al., 2013; Kisseleva, 2017). It is not expected that this issue will be settled indisputably as the lineage tracing technique, on which most studies rely to tackle the derivation of liver myofibroblast, constantly evolves with improved sensitivity and specificity.

Vascular endothelial cells form a monolayer lining the vasculature throughout the body. Specifically, liver sinusoidal endothelial cells (LSECs) are tucked between hepatocytes and HSCs. Physically a barrier between the circulation and the basal laminae, endothelial cells possess diverse roles maintaining the internal homeostasis. Multiple mechanisms have been proposed for the regulation of liver fibrosis by endothelial cells (Poisson et al., 2017). Recently, fate-mapping experiments have found that a small yet significant fraction of LSECs may directly contribute to liver fibrosis via a process known as endothelial-mesenchymal process (EndMT). EndMT is a conserved process absolutely essential for embryogenesis (Lin et al., 2012). Aberrant activation of EndMT has been demonstrated to promote fibrogenesis, to various extents, in different tissues and organs (Wynn, 2008). Transforming growth factor (TGF-β) is one of the most potent stimulators of EndMT both *in vitro* and *in vivo* (Pardali et al., 2017).

Reactive oxygen species (ROS) play a wide range of physiological and pathophysiological roles to program embryonic development and regulate postnatal life activities. ROS production is catalyzed through biochemical reactions by a panel of enzymes. NAPDH oxidase (NOX) family of proteins, consisting of both catalytic and regulatory/organizational subunits, constitute one of the major sources for ROS production. NOX4 is preferentially expressed in the vasculature with high levels of expression detected in endothelial cells (Bedard and Krause, 2007). NOX4 transcription can be activated by TGF-β in cultured endothelial cells (Hu et al., 2005; Bai et al., 2014; Yan et al., 2014). In addition, NOX4 mediated ROS production seems to serve as a permissive step toward to EndMT and tissue fibrosis (Hecker et al., 2009). The underlying epigenetic mechanism whereby TGF-β activates NOX4 transcription to promote EndMT and liver fibrosis, however, remains incompletely understood.

Brahma related gene 1 (Brg1) is a component of the mammalian SWI/SNF chromatin remodeling complex. The requirement for BRG1 in mammalian vasculogenesis has been highlighted by a series of reports providing evidence to show that BRG1 integrates multiple signaling pathways to regulate endothelial differentiation (Griffin et al., 2008, 2011; Davis et al., 2013). Surprisingly, Brg1 is non-essential for angiogenesis in several different animal models (Wiley et al., 2015). There has been scarce information regarding the role of Brg1 in regulating endothelial disorders to promote disease pathogenesis in animal models. We have previously shown that endothelial specific deletion of Brg1 protects the mice from cardiac ischemia-reperfusion injury and abdominal aortic aneurysm owing to reduced pro-inflammatory response (Zhang et al., 2018a,b). Here we report that endothelial Brg1 is essential for bile dut ligation (BDL) induced liver fibrosis in mice, which can be attributed to, at least in part, by promoting ROS-dependent EndMT. Brg1 regulates EndMT by epigenetically activating NOX4 transcription. Therefore, targeting Brg1 might yield novel therapeutic solutions against liver fibrosis.

## Materials and Methods

### Animals

All animal experiments were reviewed and approved by the Intramural Ethics Committee on Humane Treatment of Experimental Animals. All mice were bred at the Nanjing Biomedical Research Institute of Nanjing University (NBRI). Endothelial-specific deletion of BRG1 was achieved by crossing the *Smarca4*^f/f^ strain (Li et al., 2018c) to the *Cdh5*-Cre strain (Li et al., 2018e). The *Smarca4*^f/f^; *Cdh5*-Cre mice were referred to as ecKO mice and the *Smarca4*^f/f^ control mice were referred to as WT mice. Liver fibrosis was induced by bile duct ligation (BDL) as previously described (Li et al., 2018a). The mice were sacrificed 2 weeks after the surgery.

### Cell Culture, Plasmids, Transfection, and Reporter Assay

Human immortalized vascular endothelial cells (EAhy926) were maintained in DMEM supplemented with 10% FBS. Primary mouse LSECs were isolated and characterized as previously described (Meyer et al., 2016). Briefly, mice were anesthetized with isoflurane. Following perfusion and digestion, the liver suspension was passed through a 70 μm cell strainer. The non-parenchymal cells were isolated by density gradient centrifugation. LSECs were further purified by selective adherence for exactly 8 min. BRG1 expression constructs, NOX4 expression construct, and NOX4 promoter-luciferase constructs have been previously described (Ago et al., 2010; Bai et al., 2014; Li et al., 2018b). Small interfering RNAs were purchased from Dharmacon. Transient transfection was performed with Lipofectamine 2000. Cells were harvested 48 h after transfection and reporter activity was measured using a luciferase reporter assay system (Promega) as previously described (Yang et al., 2018).

### Protein Extraction and Western Blot

Whole cell lysates were obtained by re-suspending cell pellets in RIPA buffer (50 mM Tris pH7.4, 150 mM NaCl, 1% Triton X-100) with freshly added protease inhibitor (Roche) as previously described (Liu et al., 2018). Western blot analyses were performed with anti-BRG1 (Santa Cruz, sc-10768), anti-collagen type I (Rockland, 600-401-103), anti-α-SMA (Sigma, A2547), anti-VE-Cadherin (Cell Signaling Technology, 2158), anti-PECAM1 (Proteintech, 11265-1), anti-NOX4 (Proteintech, 14347-1), and anti-β-actin (Sigma, A2228) antibodies.

### RNA Isolation and Real-Time PCR

RNA was extracted with the RNeasy RNA isolation kit (Qiagen). Reverse transcriptase reactions were performed using a SuperScript First-strand Synthesis System (Invitrogen). Real-time PCR reactions were performed on an ABI Prism 7500 system. Primers and Taqman probes used for real-time reactions were purchased from Sangon Biotech (Shanghai, China).

### Chromatin Immunoprecipitation (ChIP)

Chromatin Immunoprecipitation (ChIP) assays were performed essentially as described before (Li et al., 2018d; Li Z. et al., 2019; Shao et al., 2019; Weng et al., 2019; Yang et al., 2019). In brief, chromatin in control and treated cells were cross-linked with 1% formaldehyde. Cells were incubated in lysis buffer (150 mM NaCl, 25 mM Tris pH 7.5, 1% Triton X-100, 0.1% SDS, 0.5% deoxycholate) supplemented with protease inhibitor tablet and PMSF. DNA was fragmented into ∼200 bp pieces using a Branson 250 sonicator. Aliquots of lysates containing 200 μg of protein were used for each immunoprecipitation reaction with anti-BRG1 (Santa Cruz, sc-10768), anti-p300 (Santa Cruz, sc-585), anti-c-Jun (Santa Cruz, sc-1694), anti-Fos (Santa Cruz, sc-166940), anti-SMAD3 (Abcam, ab28379), anti-ASH2 (Bethyl Laboratories, A300-489A), anti-JMJD2B (Bethyl Laboratories, A301-478), anti-anti-acetyl H3 (Millipore, 06-599), anti-trimethyl H3K4 (Millipore, 07-449), anti-trimethyl H3K9 (Millipore, 07-441), or pre-immune IgG. For re-ChIP, immune complexes were eluted with the elution buffer (1% SDS, 100 mM NaCO3), diluted with the re-ChIP buffer (1% Triton X-100, 2 mM EDTA, 150 mM NaCl, 20 mM Tris pH 8.1), and subject to immunoprecipitation with a second antibody of interest.

### DHE and DCFH-DA Staining

DHE and DCFH-DA stainings were performed essentially as previously described (Yu et al., 2018). Frozen liver sections or primary hepatocytes were stained with DHE (10 μM) or DCFH-DA (10 μM) at 37°C for 30 min. Fluorescence was visualized by con-focal microscopy (LSM 710, Zeiss). Quantifications were performed with ImageJ.

### Luminescence ROS Assay

Quantitative measurements of intracellular ROS were performed with a ROS-Glo system (Promega). Briefly, a luminescence substrate solution was added to and incubated with cultured cells for 6 h followed by the addition of the diction solution. Luminescence was measured using a microplate reader. Data were expressed as relative ROS levels compared to the control group.

### Histology

Histologic analyses were performed essentially as described before (Li N. et al., 2019). Briefly, paraffin-embedded sections were stained with picrosirius red (Sigma-Aldrich) or Masson’s trichrome (Sigma-Aldrich) according to standard procedures. Pictures were taken using an Olympus IX-70 microscope (Olympus, Tokyo, Japan).

### Statistical Analysis

One-way ANOVA with *post hoc* Scheff’e analyses were performed by SPSS software (IBM SPSS v18.0, Chicago, IL, United States). Unless otherwise specified, values of *p*<0.05 were considered statistically significant.

## Results

### Endothelial Brg1 Deficiency Attenuates BDL-Induced Liver Fibrosis in Mice

**FIGURE 1:**
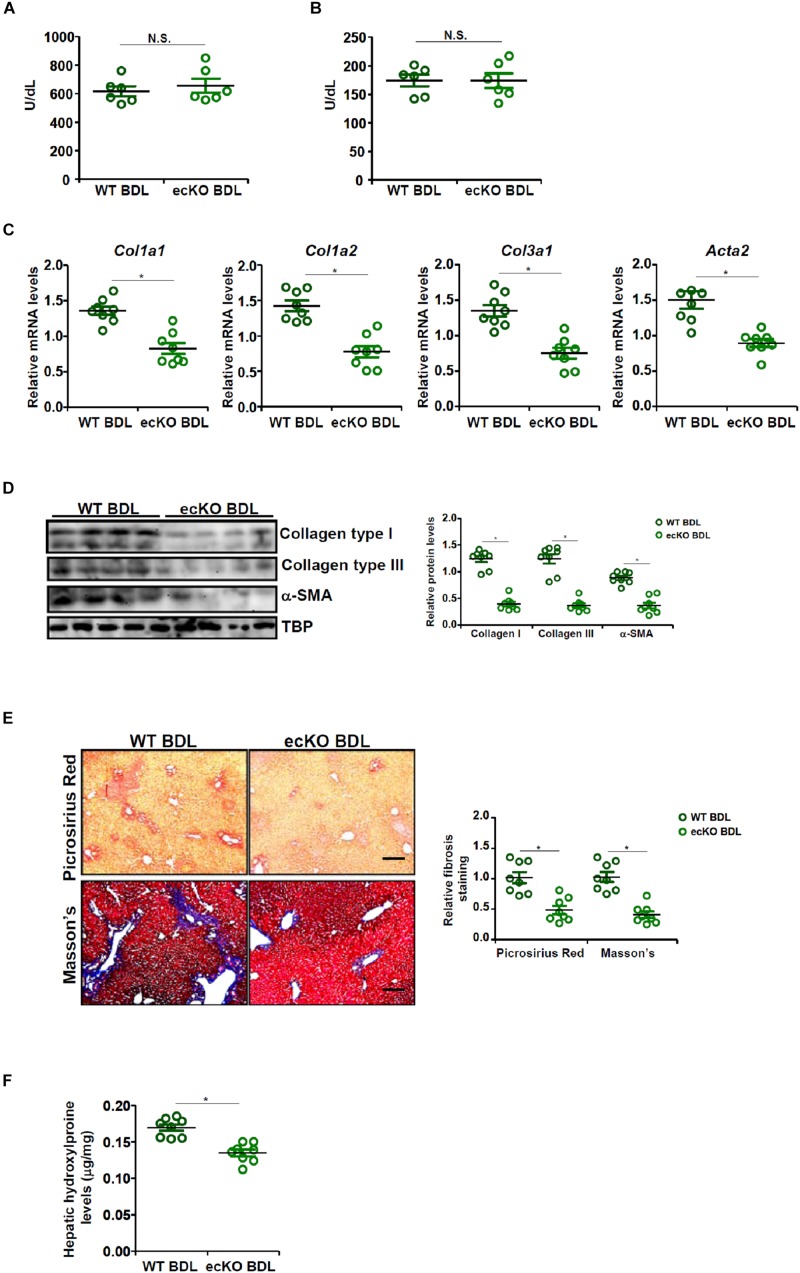
Endothelial Brg1 deficiency attenuates BDL-induced liver fibrosis in mice. Liver fibrosis was induced in endothelial-specific Brg1 knockout mice (ecKO) and wild type (WT) mice by BDL. **(A)** Plasma ALT levels. **(B)** Plasma AST levels. **(C,D)** Expression levels of pro-fibrogenic genes were examined by qPCR and Western. **(E)** Paraffin sections were stained with picrosirius red and Masson’s trichrome. Scale bar, 100 μm. **(F)** Hepatic hydroxylproline levels. *N* = 8 mice for each group. ^∗^*p* < 0.05.

The effect of endothelial-restricted Brg1 deficiency (ecKO) on liver fibrosis was evaluated in a classical animal model in which the mice were subjected to bile duct ligation (BDL) for 2 weeks. BDL procedure elicited comparable liver injury in ecKO mice and wild type (WT) littermates as assessed by plasma ALT (Figure 1A) and AST (Figure 1B) levels. Quantitative PCR (Figure 1C) and Western blotting (Figure 1D) showed that hepatic expression levels of pro-fibrogenic genes, including collagen type I, collagen type III, and alpha smooth muscle actin, were collectively down-regulated in ecKO mice compared to WT mice, suggesting that endothelial Brg1 may play a role in liver fibrogenesis. Picrosirius red staining and Masson’s trichrome staining further confirmed that there were fewer depositions of fibrillar collagens in the ecKO livers than in the WT livers (Figure 1E). Finally, hepatic hydroxylproline quantification provided additional evidence for the hypothesis that endothelial Brg1 may play a role in liver fibrosis (Figure 1F). Together, these data argue that endothelial Brg1 is essential for liver fibrogenesis.

### Brg1 Regulates Endothelial-Mesenchymal Transition *in vivo* and *in vitro*

**FIGURE 2:**
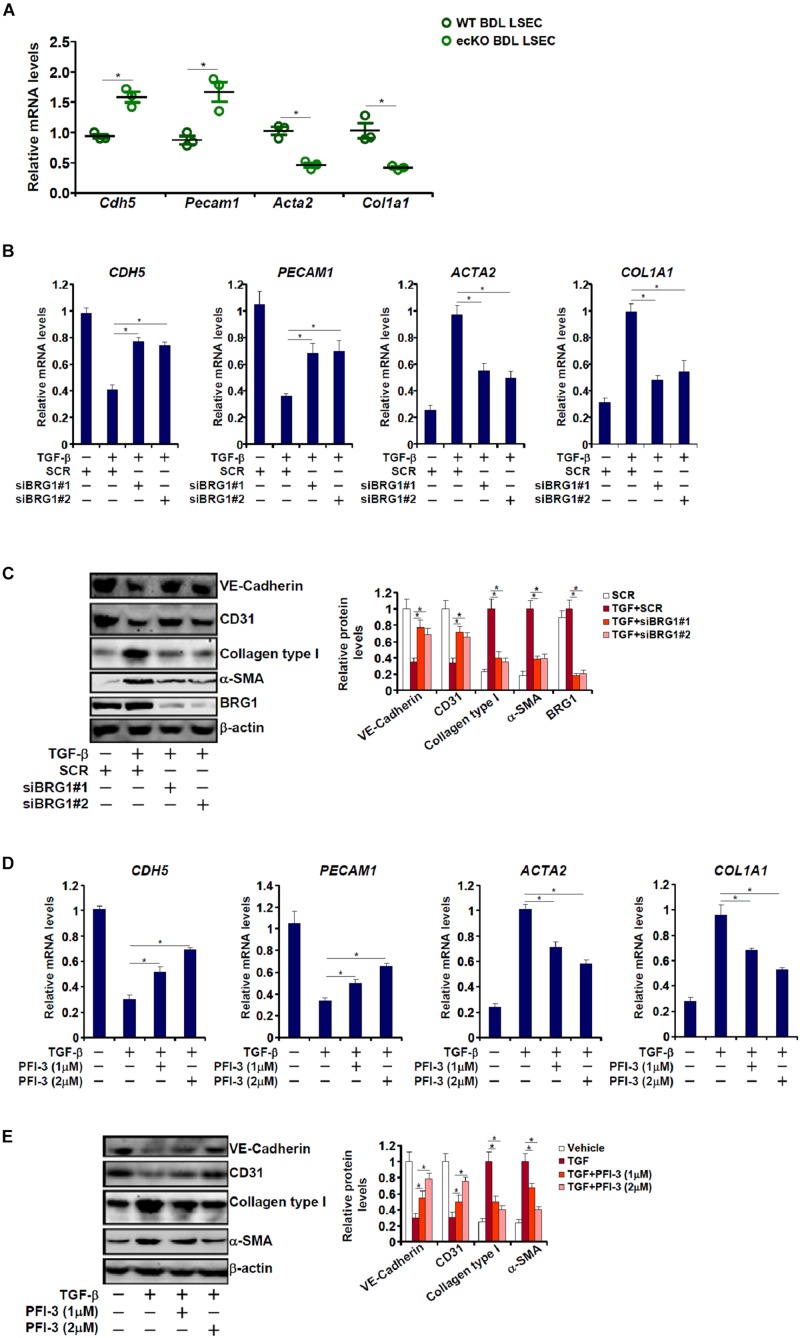
Brg1 regulates endothelial-mesenchymal transition *in vivo* and *in vitro*. **(A)** Primary LSECs were isolated from WT and ecKO mice subjected to BDL. Gene expression levels were examined by qPCR. *N* = 3 mice for each group. **(B,C)** EAhy926 cells were transfected with siRNA targeting Brg1 or SCR followed by treatment with TGF-β. Gene expression levels were examined by qPCR and Western. **(D,E)** EAhy926 cells were treated with TGF-β and/or PFI-3. Gene expression levels were examined by qPCR and Western. ^∗^*p* < 0.05.

Endothelial-mesenchymal transition (EndMT) is known to contribute to tissue fibrosis (Zeisberg et al., 2007; Wynn, 2008; Ribera et al., 2017). In light of the finding that endothelial Brg1 deficiency attenuates liver fibrosis, we asked whether Brg1 may contribute to EndMT. To this end, primary LSECs were isolated from WT or ecKO mice subjected to BDL for qPCR analysis of gene expression levels. As shown in Figure 2A, LSECs from ecKO mice exhibited higher levels of endothelial markers including Pecam1 (encoding CD31) and Cdh5 (encoding VE-Cadherin) compared to those from WT mice; there was also a simultaneous down-regulation of mesenchymal markers including Col1a1 (encoding collagen type I) and Acta2 (encoding alpha smooth muscle actin) in the LSECs from ecKO mice as opposed to the WT LESCs. We then evaluated the role of Brg1 in TGF-β induced EndMT in cultured endothelial cells. As shown in Figures 2B,C, TGF-β treatment suppressed the expression of endothelial markers (*PECAM1* and *CDH5*) and stimulated the expression of mesenchymal markers (*COL1A1* and *ACTA2*) suggestive of an EndMT-like process; two separate pairs of BRG1-targeting siRNAs comparably down-regulated Brg1 and concomitantly reversed TGF-β induced EndMT. Similarly, Brg1 inhibition by a small-molecule compound (PFI-3) dose-dependently attenuated TGF-β induced EndMT as evidenced by an up-regulation of endothelial marker genes and a simultaneous down-regulation of mesenchymal marker genes (Figures 2D,E).

### Regulation of EndMT by Brg1 Depends on NOX4 Expression

**FIGURE 3:**
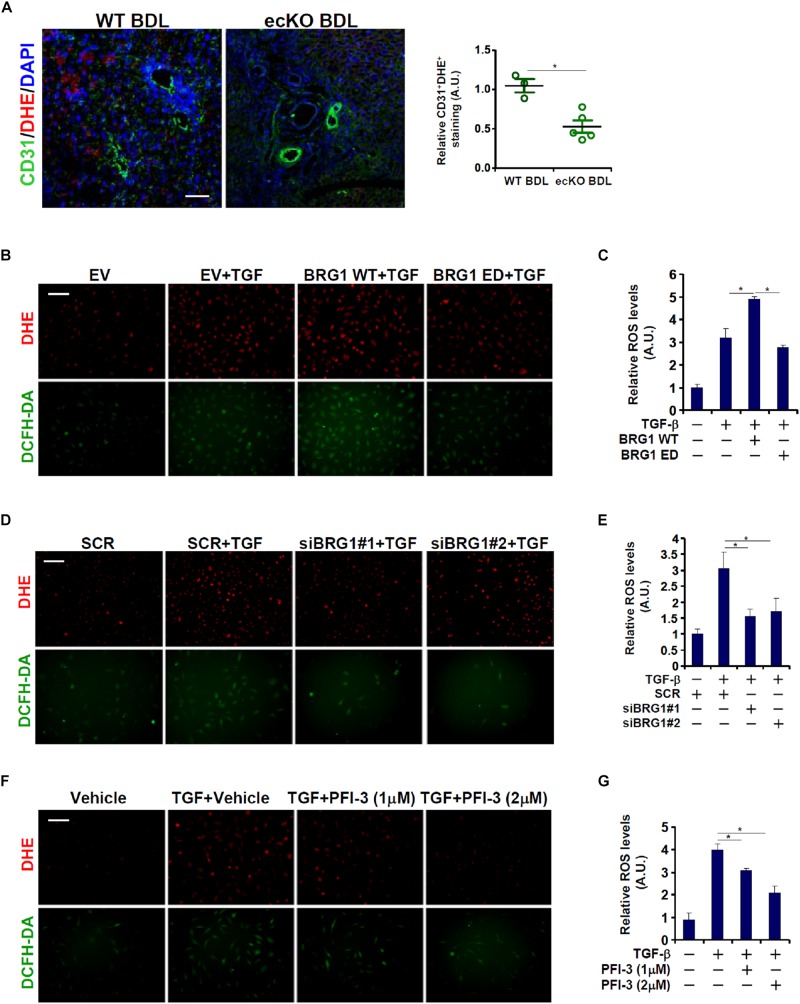
Brg1 deficiency attenuates ROS production in endothelial cells *in vivo* and *in vitro*. **(A)** Liver fibrosis was induced in WT mice and ecKO mice by BDL. ROS production in endothelial cells was verified by immunofluorescence/DHE staining. Scale bar, 100 μm. *N* = 3∼5 mice for each group. **(B,C)** EAhy926 cells were transfected with Brg1 followed by treatment with TGF-β. ROS levels were examined by DHE/DCFH-DA staining and luminescence assay. Scale bar, 100 μm. **(D,E)** EAhy926 cells were transfected with siRNA targeting Brg1 or SCR followed by treatment with TGF-β. ROS levels were examined by DHE/DCFH-DA staining and luminescence assay. Scale bar, 100 μm. **(F,G)** EAhy926 cells were treated with TGF-β and/or PFI-3. ROS levels were examined by DHE/DCFH-DA staining and luminescence assay. Scale bar, 100 μm. *N* = 3 for all the *in vitro* experiments. ^∗^*p* < 0.05.

A connection between Brg1 and ROS production has been demonstrated in various cell types (Li et al., 2018e; Liu et al., 2019). Since ROS appears to play an essential role in EndMT (Thuan et al., 2018), we asked whether Brg1 could regulate endothelial ROS levels in the context of EndMT and liver fibrosis. Immunofluorescence staining coupled with DHE staining showed that there was a significant reduction in ROS levels in endothelial cells (CD31^+^) in the ecKO livers compared to the WT livers following BDL (Figure 3A). In cultured cells, TGF-β treatment induced robust ROS production as assessed by DHE and DCHF-DA stainings (Figure 3B) and luminescence assay (Figure 3C); over-expression of wild type (WT) Brg1, but not enzyme deficient (ED) Brg1, further enhanced TGF-β induced ROS levels. On the contrary, Brg1 depletion (Figures 3D,E) or pharmaceutical inhibition (Figures 3F,G) suppressed TGF-β induced ROS levels, suggesting that Brg1 may indeed drive endothelial ROS production both *in vivo* and *in vitro*.

**FIGURE 4:**
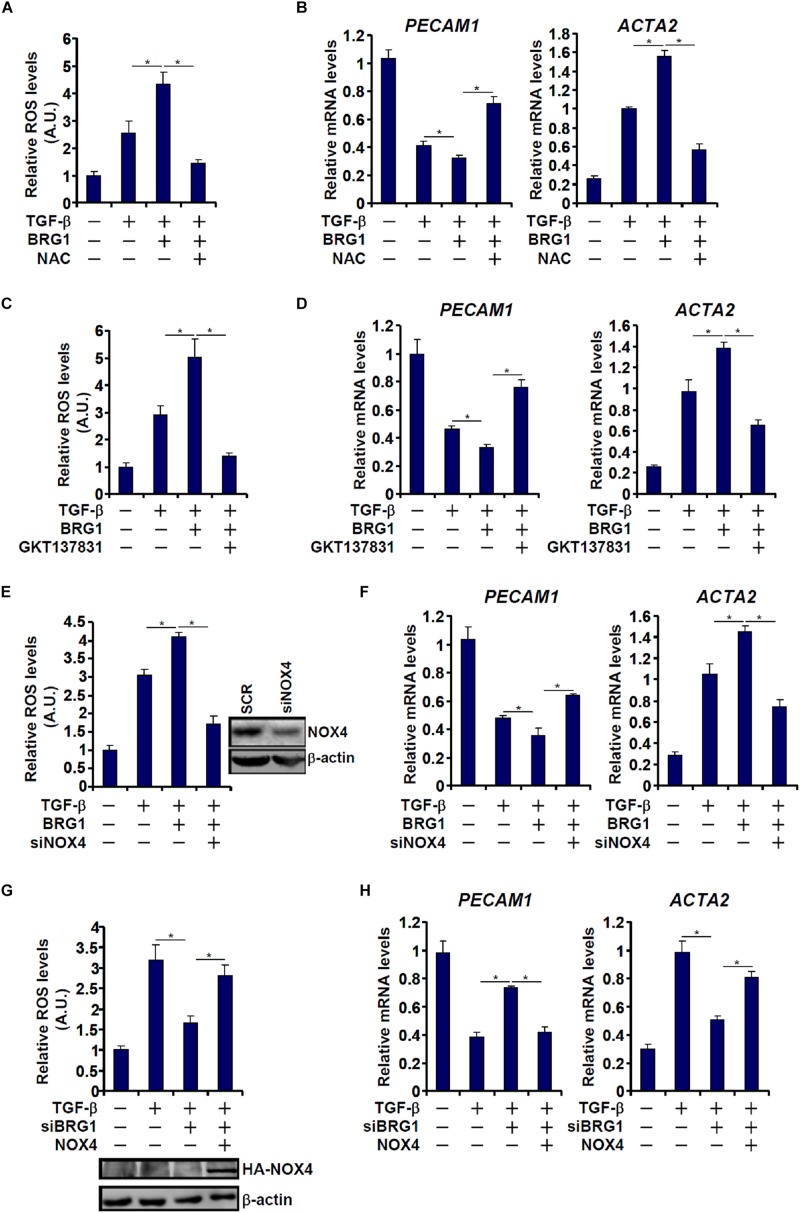
Regulation of EndMT by Brg1 depends on NOX4 expression. **(A,B)** EAhy926 cells were transfected with BRG1 followed by treatment with TGF-β in the presence or absence of NAC. ROS levels were examined by luminescence assay. Gene expression levels were examined by qPCR. **(C,D)** EAhy926 cells were transfected with BRG1 followed by treatment with TGF-β in the presence or absence of siRNA targeting NOX4. ROS levels were examined by luminescence assay. Gene expression levels were examined by qPCR. **(E,F)** EAhy926 cells were transfected with Brg1 followed by treatment with TGF-β in the presence or absence of GKT. ROS levels were examined by luminescence assay. Gene expression levels were examined by qPCR. **(G,H)** EAhy926 cells were transfected with siRNA targeting Brg1 or SCR followed by treatment with TGF-β in the presence or absence of NOX4. ROS levels were examined by luminescence assay. Gene expression levels were examined by qPCR. *N* = 3 for all the *in vitro* experiments. ^∗^*p* < 0.05.

NAPDH oxidase 4 (NOX4) is one of the major NOX isoforms and preferentially expressed in endothelial cells. There is evidence to suggest that NOX4 is involved in EMT and liver fibrosis (Lan et al., 2015). We asked whether there might be co-dependence between Brg1 and NOX4 in regulating EndMT. TGF-β treatment stimulated ROS production (Figure 4A) and EndMT (Figure 4B) in endothelial cells; Brg1 over-expression enhanced the TGF effects, both of which were reversed by the addition of NAC, an antioxidant. Similarly, the ability of Brg1 to augment TGF-induced ROS generation and EndMT was blocked by either pharmaceutical inhibition of NOX4 with a small-molecule compound (GKT137831, Figures 4C,D) or depletion of NOX4 expression with siRNA (Figures 4E,F). On the contrary, whereas Brg1 knockdown suppressed TGF-induced ROS synthesis (Figure 4G) and EndMT (Figure 4H), forced NOX4 expression circumvented Brg1 deficiency by restoring ROS levels to allow EndMT to proceed.

### Brg1 Is Essential for NOX4 Expression *in vivo* and *in vitro*

**FIGURE 5:**
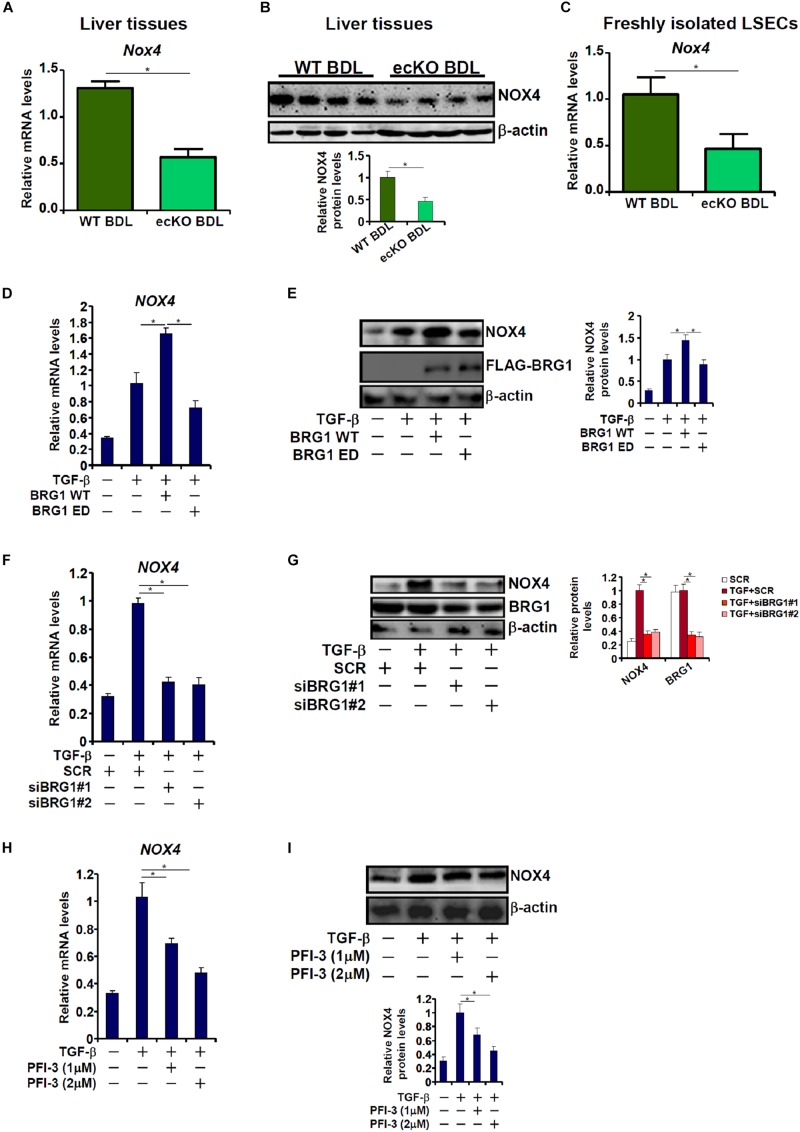
Brg1 is essential for NOX4 expression *in vivo* and *in vitro*. **(A,B)** WT and ecKO mice were subjected to BDL. NOX4 expression was examined by qPCR and Western. *N* = 6 mice for each group. **(C)** Primary LSECs were isolated from WT and ecKO mice subjected to BDL. NOX4 expression was examined by qPCR. *N* = 3 mice for each group. **(D,E)** EAhy926 cells were transfected with WT or ED Brg1 followed by treatment with TGF-β. NOX4 expression was examined by qPCR and Western. **(F,G)** EAhy926 cells were transfected with siRNA targeting Brg1 or SCR followed by treatment with TGF-β. NOX4 expression was examined by qPCR and Western. **(H,I)** EAhy926 cells were treated with TGF-β in the presence or absence of PFI-3. NOX4 expression was examined by qPCR and Western. *N* = 3 for all the *in vitro* experiments. ^∗^*p* < 0.05.

The reliance of Brg1 on NOX4 to regulate ROS and EndMT raised the possibility that Brg1 may contribute to NOX4 expression in endothelial cells. Quantitative PCR (Figure 5A) and Western blotting (Figure 5B) suggested a reduction of NOX4 expression in the ecKO livers compared to the WT livers following BDL. In addition, LSECs freshly isolated from ecKO mice exhibited lower NOX4 expression than those isolated from WT mice (Figure 5C). In cultured endothelial cells, Brg1 over-expression significantly enhanced NOX4 induction by TGF-β treatment (Figures 5D,E). In contrast, Brg1 knockdown or Brg1 inhibition dampened NOX4 induction by TGF-β treatment (Figures 5F,G). Brg1 inhibition by PFI-3 also ameliorated NOX4 induction (Figures 5F–I).

### Brg1 Activates NOX4 Transcription by Interacting With AP-1 and SMAD

**FIGURE 6:**
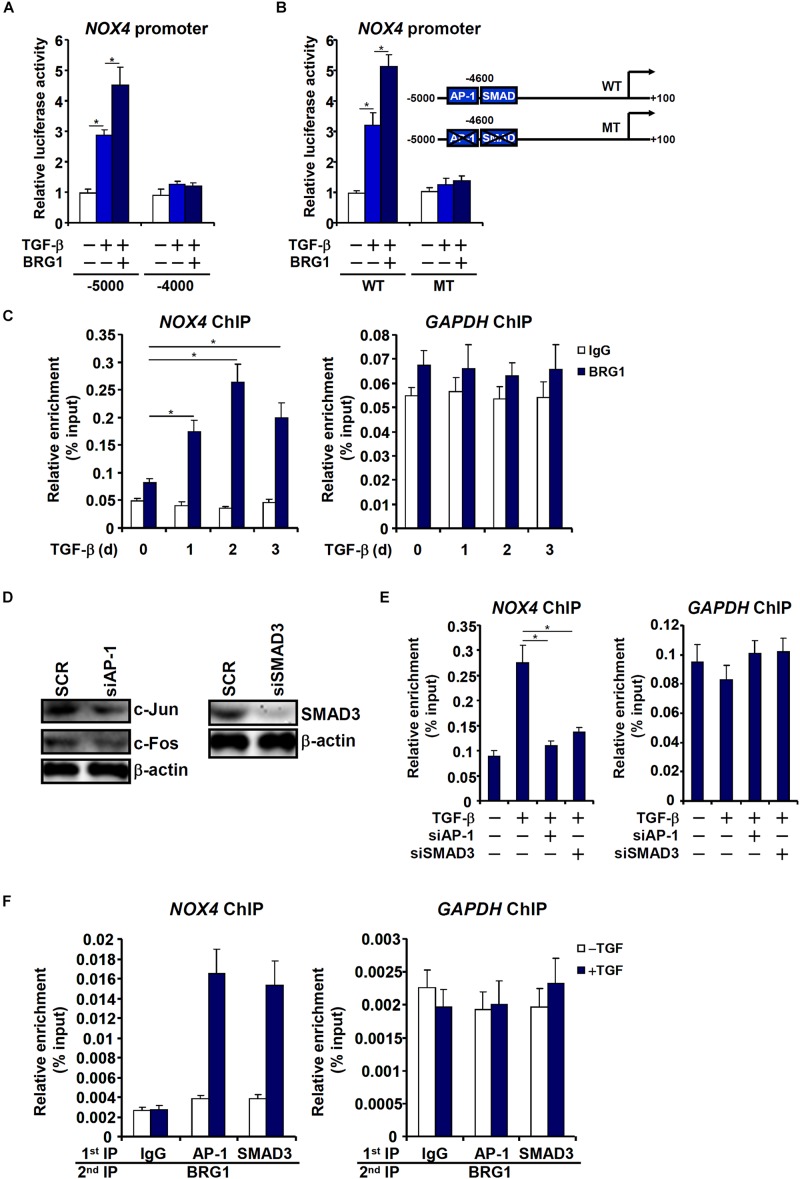
Brg1 activates NOX4 transcription by interacting with AP-1 and SMAD. **(A)** Different NOX4 promoter-luciferase constructs were transfected into EAhy926 cells with or without Brg1 followed by treatment with TGF-β. Luciferase activities were normalized by both protein concentration and GFP fluorescence. **(B)** Wild type (WT) or mutant (MT) NOX4 promoter-luciferase constructs were transfected into EAhy926 cells with or without Brg1 followed by treatment with TGF-β. Luciferase activities were normalized by both protein concentration and GFP fluorescence. **(C)** EAhy926 cells were treated with TGF-β and harvested at indicated time points. ChIP assays were performed with anti-Brg1. **(D,E)** EAhy926 cells were transfected with indicated siRNAs followed by treatment with TGF-β. Knockdown efficiencies were examined by Western. ChIP assays were performed with anti-BRG1. **(F)** EAhy926 cells were treated with or without TGF-β for 48 h. Re-ChIP assays were performed with indicated antibodies. *N* = 3 for all the *in vitro* experiments. ^∗^*p* < 0.05.

Having determined that Brg1 is essential for NOX4 expression, we sought to uncover the mechanism whereby Brg1 activates NOX4 transcription. Reporter assay indicated that Brg1 over-expression potentiated induction of an NOX4 promoter (∼5,000 bp relative to the transcription start site) by TGF-β (Figure 6A). However, neither TGF-β treatment nor Brg1 over-expression influenced a shorter NOX4 promoter with an additional inward deletion of 1,000 bp. A distal TGF response element (TRE) containing two adjacent binding sites for AP-1 and SMAD3 respectively has been mapped to the distal NOX4 promoter (Bai et al., 2014). Mutation of the distal TRE not only abrogated TGF induction but abolished BRG1 potentiation (Figure 6B). ChIP assay confirmed that Brg1 was recruited to the NOX4 promoter, but not the GAPDH promoter, by TGF-β stimulation peaking at 24 h (Figure 6C). Depletion of either AP-1 or SMAD3 (Figure 6D) severely disturbed Brg1 binding to the NOX4 promoter (Figure 6E), suggesting that Brg1 may rely on both AP-1 and SMAD3 for NOX4 promoter. Indeed, Re-ChIP assay showed that TGF treatment strongly promoted the interaction between Brg1 and AP-1/SMAD3 on the NOX4 promoter (Figure 6F).

### Brg1 Regulates NOX4 Transcription by Recruiting Histone Modifying Enzymes

**FIGURE 7:**
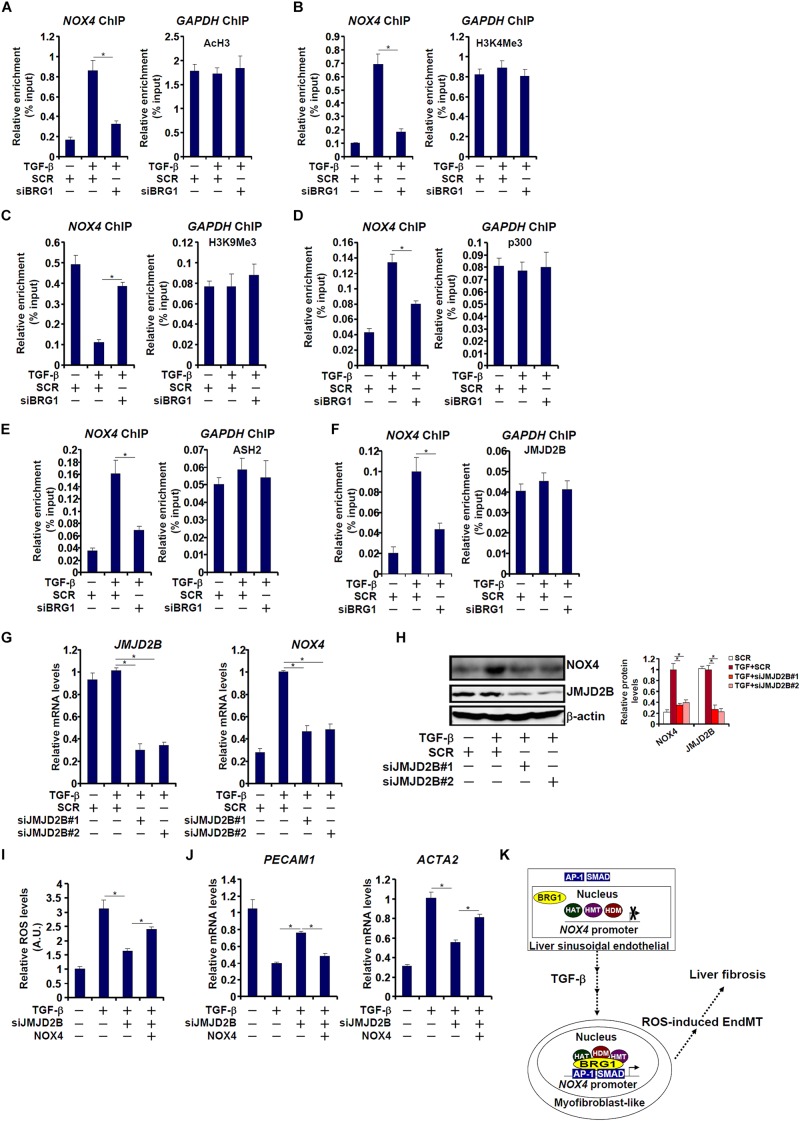
Brg1 regulates NOX4 transcription by recruiting histone modifying enzymes. **(A–F)** EAhy926 cells were transfected with siRNA targeting Brg1 or SCR followed by treatment with TGF-β. ChIP assays were performed with indicated antibodies. **(G,H)** EAhy926 cells were transfected with siRNA targeting JMJD2B or SCR followed by treatment with TGF-β. NOX4 expression was examined by qPCR and Western. **(I,J)** EAhy926 cells were transfected with siRNA targeting BRG1 or SCR followed by treatment with TGF-β in the presence or absence of NOX4. ROS levels were examined by luminescence assay. Gene expression levels were examined by qPCR. *N* = 3 for all the *in vitro* experiments. **(K)** A schematic model. ^∗^*p* < 0.05.

We next investigated the epigenetic mechanism whereby Brg1 regulates NOX4 transcription in endothelial cells. ChIP assays showed that TGF-β treatment evoked robust accumulation of active histone markers such as acetyl H3 (Figure 7A) and trimethyl H3K4 (Figure 7B) on the NOX4 promoter. In the meantime, trimethyl H3K9, a repressive histone marker, was erased from the NOX4 promoter by TGF-β treatment (Figure 7C). These changes, indicative of an open chromatin structure, were consistent with NOX4 trans-activation. Brg1 knockdown, however, suppressed acetyl H3 and trimethyl H3K4 but restored trimethyl H3K9 (Figures 7A–C). In keeping with the alterations of histone modifications, ChIP assays further revealed that p300, a histone acetyltransferase (Figure 7D), ASH2, a key regulatory subunit of the H3K4 methyltransferase complex (Figure 7E), and JMJD2B, an H3K9 tri-demethyalse (Figure 7F), were all recruited to the NOX4 promoter by TGF-β stimulation. Brg1 depletion significantly dampened the recruitment of all three histone modifying enzymes, suggesting that activation of NOX4 transcription by Brg1 may be mediated through differential histone modifications.

We then further investigated the role of JMJD2B in TGF-β induced NOX4 transcription and EndMT. As shown in Figures 7G,H, JMJD2B silencing by siRNAs attenuated TGF-β induced NOX4 expression. As a result, intracellular ROS levels (Figure 7I) were down-regulated, which probably led to a partial reversion of EndMT (Figure 7J). Re-introduction of ectopic NOX4, however, bypassed the requirement of JMJD2B to restore intracellular ROS levels (Figure 7I) and permit EndMT to proceed (Figure 7J).

## Discussion

Reactive oxygen species (ROS) play critical roles to steer vascular endothelial cell differentiation during organ development (Zhou et al., 2013). However, excessive ROS production in endothelial cells is usually associated with disruption of endothelial function and heralds the pathogenesis of a host of diseases in humans. For instance, NOX4-catalyzed ROS production in endothelial cells has been shown to promote tissue fibrosis likely via pivoting endothelial cells toward a myofibroblasts-like phenotype in a process known as EndMT (Hecker et al., 2009). Here we show that the chromatin remodeling protein Brg1 serves as a “switch” for that process by activating NOX4 transcription. Accordingly, endothelial-specific Brg1 deficiency is paralleled by attenuation of EndMT and, consequently, regression of liver fibrosis (Figure 7K). Mounting evidence suggests that BRG1 plays indispensible roles defining the identity of vascular endothelial cells during embryogenesis. Accordingly, targeted disturbance of BRG1 expression in endothelial cells, either constitutively or inducibly, causes developmental arrest with deformation of vasculature throughout the body (Stankunas et al., 2008; Menendez et al., 2017; Vieira et al., 2017). Notably, Brg1 appears to be non-essential for endothelial cells to maintain a differentiated state and normal function under physiological conditions (Wiley et al., 2015). Therefore we propose that Brg1 may act as a stress protein that integrates injurious cues to program endothelial dysfunction. This notion, awaiting further verification, portrays Brg1 as an attractive target for intervention as normal cells are unlikely influenced by the loss of Brg1 thus avoiding unintended side effects.

Although we show that Brg1 promotes EndMT and liver fibrosis through regulating NOX4 transcription and ROS production in endothelial cells, other possibilities cannot be excluded. EndMT shares many regulatory molecules with epithelial-mesenchymal transition (EMT). For instance, the Snail family of transcriptional repressors, including Snail, Zeb, and Twist, bind to the promoter regions and repress transcription of epithelial/endothelial marker genes (e.g., VE-Cadherin) (Lamouille et al., 2014). It has been shown that Brg1 is capable of acting as a co-factor for Zeb1 to promote EMT and metastasis in a number of different cancer cells (Sanchez-Tillo et al., 2010). Alternatively, Brg1 has been found to directly responsible for activating the transcription of Snail2/Slug (Eroglu et al., 2006) and Twist (Huang et al., 2018). In addition, Brg1 has been implicated as an important mediator for several key signaling pathways involved in EndMT. For instance, Ross et al. have shown that Brg1 potentiates the TGF signaling, one of the most potent stimulators of EndMT, by acting as a co-factor for SMAD (Ross et al., 2006). Brg1 has also been shown to participate in the Wnt pathway by controlling the bioavailability of the signaling molecules (e.g., ligands, receptors, signaling adaptors) and by directly activating β-catenin target genes (Griffin et al., 2011). Of note, ROS levels are intimately associated with the TGF signaling pathway (Liu and Desai, 2015) and the Wnt signaling pathway (Korswagen, 2006). These observations, although not yet authenticated in the current setting, highlight the potential of Brg1 as a versatile regulator of EndMT. Clearly, these alternative scenarios are worth further investigation with a focus on Brg1 being a possible bridge between ROS status and EndMT.

We report here that Brg1 may rely on its interaction with the histone acetyltransferase p300 to activate NOX4 transcription and ROS production. Leto and colleagues have previously shown that mutant p53 drives NOX4 transcription in a range of different cancer cells via recruiting p300 to acetylate histone H4K8 surrounding the NOX4 promoter (Boudreau et al., 2017). Sanders et al. (2015) and Liu et al. (2019) have previously reported that up-regulation of NOX4 transcription is accompanied by accumulation of acetylated H4K16, mediated by the acetyltransferase hMOF/MYST1, on the NOX4 promoter in lung fibroblast cells and in hepatocytes, respectively. We did not observe significant enrichment of either H4K16 or hMOF on the NOX4 promoter in endothelial cells following TGF-β treatment (data not shown), suggesting that BRG1 may recruit distinct sets of histone modifying enzymes to activate NOX4 transcription in a cell type- and stimulus-specific manner. On the other hand, the finding that recruitment of the histone demetheylase JMJD2B by BRG1 activates NOX4 transcription and ROS generation deserves further attention because no prior evidence exists to link JMJD2B to ROS regulation. ROS production can be deemed as a function/dysfunction of mitochondria, where NOX4 is located (Shanmugasundaram et al., 2017). It is noteworthy that JMJD2B has been implicated in the regulation of mitochondrial function (Sun et al., 2014; Li et al., 2016; Kang et al., 2018). Intriguingly, a recent report by Costantino et al. (2019) have revealed that there was an inverse correlation between H3K9 methylation and ROS levels in visceral fat arteries (VFAs) isolated from individuals with obesity compared to the lean subjects. Mice with germline deficiency of JMJD2B are viable but develop spontaneous obesity and type 2 diabetes (Cheng et al., 2018). It is not known at this point whether endothelial-specific JMJD2B deficiency would protect the mice from liver fibrosis, similar to the Brg1 ecKO mice. Additional studies are warranted to clarify this unresolved issue.

In summary, our data illustrate a novel epigenetic mechanism that links ROS-driven EndMT to liver fibrosis. Future investigations should put this model to further and more rigorous test before a rationalized decision can be made to target this Brg1-NOX4 axis to treat liver fibrosis.

## Data Availability Statement

The datasets generated for this study are available on request to the corresponding author.

## Ethics Statement

The animal study was reviewed and approved by Nanjing Medical University Ethics Committee on Humane Treatment of Experimental Animals.

## Author Contributions

YX, JG, and JL conceived the project. ZL, BC, WD, MK, and YX designed the experiments. ZL, BC, WD, MK, YS, ZF, LY, and DW performed the experiments, collected the data, and analyzed the data. JL and JG provided funding and supervision. YX wrote the manuscript with inputs from all authors.

## Conflict of Interest

The authors declare that the research was conducted in the absence of any commercial or financial relationships that could be construed as a potential conflict of interest.
